# New familial cases of karyomegalic interstitial nephritis with mutations in the *FAN1* gene

**DOI:** 10.1186/s12920-021-01009-7

**Published:** 2021-06-14

**Authors:** Imen Rejeb, Mouna Jerbi, Houweyda Jilani, Hanène Gaied, Yasmina Elaribi, Syrine Hizem, Raja Aoudia, Hafedh Hedri, Chiraz Zaied, Salwa Abid, Hassen Bacha, Taieb BenAbdallah, Lamia BenJemaa, Rim Goucha

**Affiliations:** 1 Service des Maladies Congénitales et Héréditaires, CHU Mongi Slim La Marsa, La Marsa, Tunisia; 2 Service de Néphrologie, CHU Mongi Slim La Marsa, La Marsa, Tunisia; 3 Laboratory of Renal Pathology LR00SP01, Tunis, Tunisia; 4 Faculty of Medicine, University Tunis El Manar, Tunis, Tunisia; 5 Department of Internal Medicine, Charles Nicolle Hospital, Tunis, Tunisia; 6 Faculty of Dentistry, Monastir, Tunisia

**Keywords:** Karyomegalic interstitial nephritis, Chronic tubulointerstitial nephritis, *FAN1* gene, Frameshift variants

## Abstract

**Background:**

Karyomegalic interstitial nephritis (KIN) is a rare disease entity first described by Burry in 1974. The term KIN was introduced by Mihatsch et al. in 1979. KIN is characterized by chronic tubulointerstitial nephritis associated with enlarged tubular epithelial cell nuclei, which leads to a progressive decline of renal function. The prevalence of this disease is less than 1% of all biopsies, and its pathogenesis is unclear. KIN results from mutations in *FAN1* (FANCD2/FANCI-Associated Nuclease 1), a gene involved in the DNA damage response pathway, particularly in the kidney. In this study, we report two Tunisian consanguineous families with KIN caused by mutations in the *FAN1* gene.

**Methods:**

Direct sequencing of the coding regions and flanking intronic sequences of the *FAN1* gene was performed in three affected members. Three prediction programs (Polyphen-2 software, SIFT, and MutationTaster) were used to predict the functional effect of the detected variations.

**Results:**

Two causative frameshift variants in the *FAN1* gene were identified in each family: The previously described frameshift mutation c.2616delA (p.Asp873ThrfsTer17) and a novel mutation c.2603delT (p.Leu868ArgfsTer22) classified as "pathogenic" according to the American College of Medical Genetics and Genomics (ACMG) guidelines.

**Conclusion:**

To our best knowledge, this is the first Tunisian study involving familial cases of KIN with mutations in the *FAN1* gene. We hypothesize that these findings can expand the mutational spectrum of KIN and provide valuable information on the genetic cause of KIN.

## Background

Karyomegalic interstitial nephritis (KIN) is a rare disease that was first described by Burry [1] and given this term later by Mihatsch et al. [2]. A history of recurrent respiratory infections and progressive renal failure was described. Since then, approximately 50 cases of KIN and 12 families have been reported in the literature [3–5].

KIN is characterized by chronic tubulointerstitial nephritis associated with enlarged tubular epithelial cell nuclei, leading to the progressive decline of renal function. Its prevalence is below 1% of all biopsies [6]. Its pathogenesis is unclear yet.

Mutations in the *FAN1* gene involved in the DNA damage response pathway, including the kidney, have been reported in 2012 as causative in familial cases of KIN [4]. These findings allowed us to highlight the potential link between defective DNA repair and chronic kidney disease progression. This is the first reported Tunisian study involving familial cases of KIN with mutations in the *FAN1* gene.

## Methods

### Case presentation

**Fig. 1:**
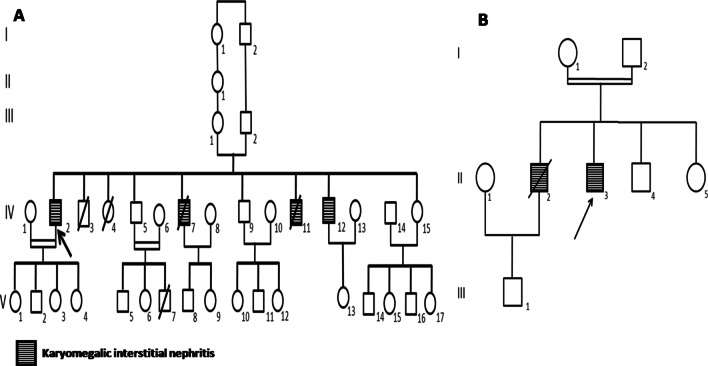
**a** Pedigree of the family A, **b** Pedigree of the family Z

Two consanguineous families from the north of Tunisia (Fig. 1) were referred to our nephrology department because of impaired renal function caused by KIN.

### Microscopy analysis

The renal biopsies of the patients were examined by light microscopy (Nikon Eclipse E400 Research Microscope) using conventional staining methods and immunofluorescence. The photos were taken using the Nikon Coolpix 4500 4MP Digital camera.

For the electron microscopy we used a Jeol 1010 transmission electron microscope operated at 80 kV.

#### Family A

##### Proband IV-2

Born in 1963, he was admitted for convulsions. A chronic renal failure linked to tubular nephropathy with hepatic cytolysis and cholestasis was suspected. Renal biopsy was not performed because of small kidneys. The diagnosis of end-stage renal disease (ESRD) due to tubulointerstitial nephritis was retained.

The serum creatinine level was 900 μmol/L, the estimated glomerular filtration rate (eGFR) was 9.9 mL/min/1.73 m^2^, and proteinuria was 0.25 g/24 h.

The patient received a living renal transplant from his wife (patient IV-1), a second-degree cousin. His renal function was still normal 18 years later.

##### Patient IV-7

Born in 1969, the disease was discovered during a kidney donor checkup. A kidney failure was detected with serum creatinine level at 161 µmol/L and eGFR at 69.4 mL/min/1.73 m^2^. Proteinuria was absent in the 24-h urine dosage.

**Fig. 2:**
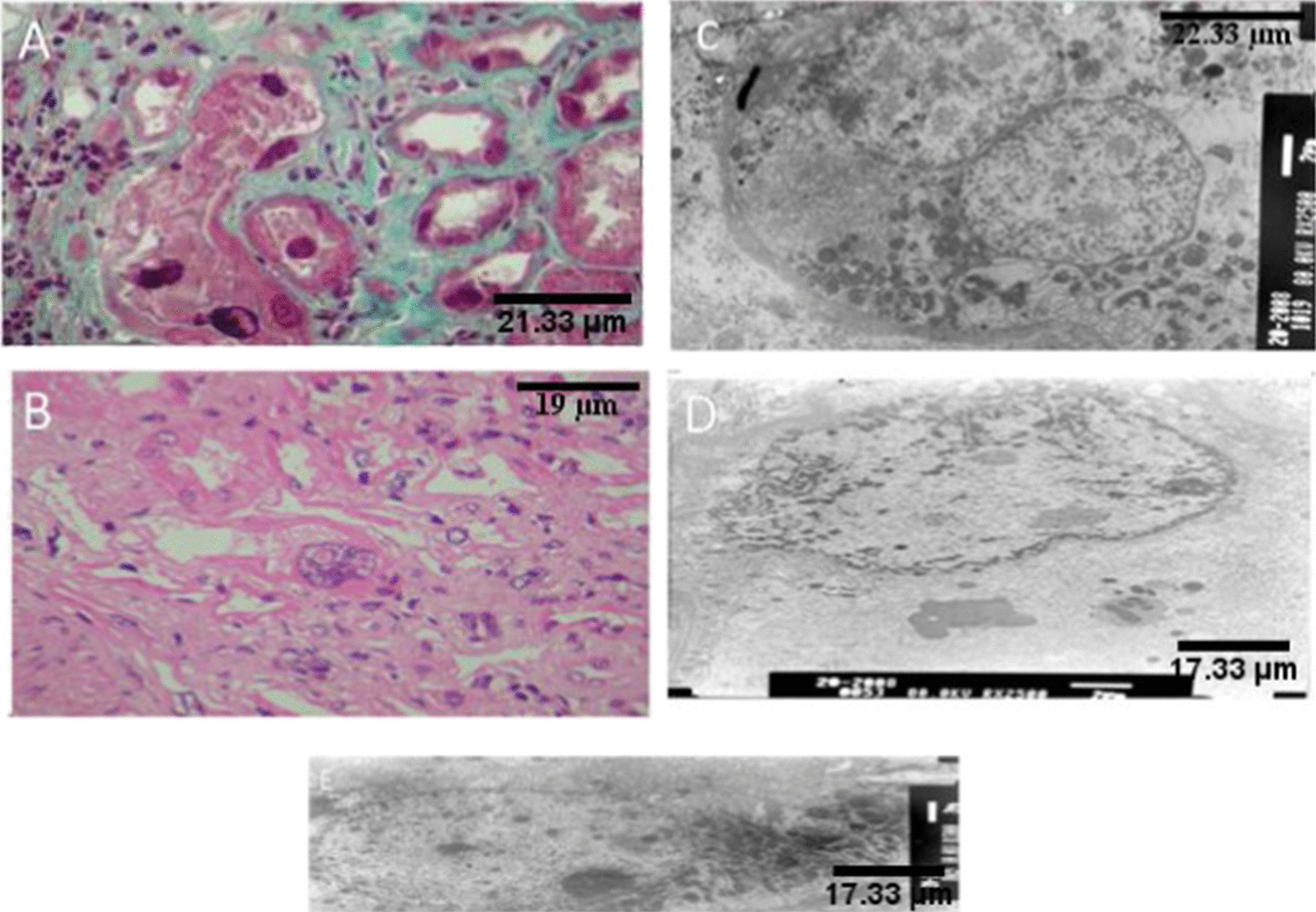
Renal histology in Patient IV-7 and Patient IV-12 in family A. **a** Renal biopsy, Masson’s trichroma (magnification × 400). Proximal tubules with giant nuclei, irregular nuclear membrane (Patient IV-7). **b** Renal biopsy, hematoxylin and eosine stain (magnification × 200). Giant nuclei with irregular nuclear membrane (Patient IV-7). **c**, **d** Renal biopsy, electron microscopy. Giant karyomegalic nuclei with irregular nuclear membrane (Patient IV-7). **e** Renal biopsy, electron microscopy. Giant karyomegalic nuclei of epithelial cells of proximal tubules (Patient IV-12)

Renal biopsy revealed KIN. The obtained renal tissue contained 21 glomeruli: 7 were sclerotic and atrophic and 14 were normal. The epithelial cells were atrophic or normal. Proximal or distal tubules contained giant nuclei with irregular nuclear membrane and rare inclusions in 1–2 nuclei. The interstitial tissue showed fibrosis with mononuclear infiltrations. Immune deposits were not detected in immunofluorescence. Electron microscopy showed giant karyomegalic nuclei in the epithelial cells of proximal tubules that were severely enlarged, polymorphic and lobulated. The nuclear membrane had intense invagination and lobulation (Fig. 2).

The patient received a living renal transplant and was deceased from pulmonary embolism.

##### Patient IV-11

Born in 1974, he consulted for a kidney donor checkup. Tubulointerstitial nephritis with chronic renal failure was diagnosed. Renal biopsy was impossible to do because of small kidneys.

Serum creatinine level was at 197 µmol/L, eGFR at 53.38 mL/min/1.73 m^2^.

The patient underwent hemodialysis for 3 years and was deceased from severe infection.

##### Patient IV-12

Born in 1976, he was admitted to the nephrology department for an incidental finding of renal failure, after the discovery of nephropathy in his two brothers (Patients IV-2 and IV-11).

Serum creatinine level was at 141 µmol/L, eGFR was at 65.9 mL/min/1.73 m^2^ and proteinuria was absent in the 24-h urine dosage.

Renal biopsy showed KIN. The obtained renal tissue contained 39 glomeruli: 13 were sclerotic and atrophic and the remaining 26 were normal. The interstitial tissue showed a fibrosis band and atrophic tubules, giant nuclei of tubular epithelium with irregular nuclear membrane. Immune deposits were not detected in immunofluorescence. Electron microscopy revealed epithelial cells of proximal tubules with karyomegalic nuclei. The nucleoli had variable size and polymorphism. The tubular basement membranes were occasionally thickened without demonstrable osmiophilic deposits (Fig. 2).

Liver function test revealed cytolysis and cholestasis in patient IV-2 and IV-12. Patient IV-7 had cholestasis. Liver biopsy performed in patient IV-7 showed no specific pathology.

Urinalysis indicated negative proteinuria, hematuria, and glycosuria for IV-2 and IV-12. In patient IV-7, urinalysis showed negative hematuria and glycosuria, and trace proteinuria. Leukocyturia 2 + was found in patients IV-2 and IV-7.

Viral serology, serum ANCA (Antineutrophil cytoplasmic antibody), ANA (Antinuclear antibody) immunofixation, and free light chain levels were within the normal range for patients IV-2, IV-7, and IV-12.

Available laboratory parameters and clinical data are summarized in Table 1.

**Table 1:** Clinical and biological findings of patients with KIN in families A and Z

	Patient No	Sexe	AN	AERSD	Smoking	Personal history	Cause of renal examination	HMG	TR	Death	Initial creatinine (µmol/l)	eGFR ml/min/1.73m^2^	Cytolisis	Cholestasis	VST	KB	Liver Biopsy
Family A	IV-2	M	36	36	No	0	Convulsion	0	Yes	No	900	9.9	Yes	Yes	–	No	No
	IV-7	M	39	43	Yes	Appendectomy	Donor check up	0	Yes	Yes	161	69.4	No	Yes	–	Yes K +	Yes K-
	IV-11	M	26	29	No	0	Donor Check up	0	No	Yes	197	53.38	Yes	Yes	–	No	No
	IV-12	M	32	38	No	Urethritis	Donor Check up	0	No	No	141	65.9	Yes	Yes	–	Yes K +	No
Family Z	II-2	M	31	36	No	0	Fortuitously	0	Yes	Yes	1000	ND	No	No	–	No	No
	II-3	M	25	33	No	Epilepsy and visceral leishmaniasis	Donor check up	0	No	No	200	ND	No	No	–	Yes K +	Yes K-

AN, age at the time of the discovery of nephropathy; AERSD, Age at the time of the end of renal stage disease; HMG, hepatomegaly; TR, transplantation; eGFR, estimated glomerular filtration rate; VST, viral serology test; KB, Kidney biopsy; K, Karyomegaly; + , present; -, absent; ND, no data

#### Family Z

The second family was also a consanguineous family from the north of Tunisia, with eight individuals who did not live in rural areas (Fig. 1b).

##### Patient II-2

He was diagnosed with ESRD at the age of 31 and treated by peritoneal dialysis. He was deceased from pulmonary tuberculosis four years later.

##### Patient II-3

**Fig. 3:**
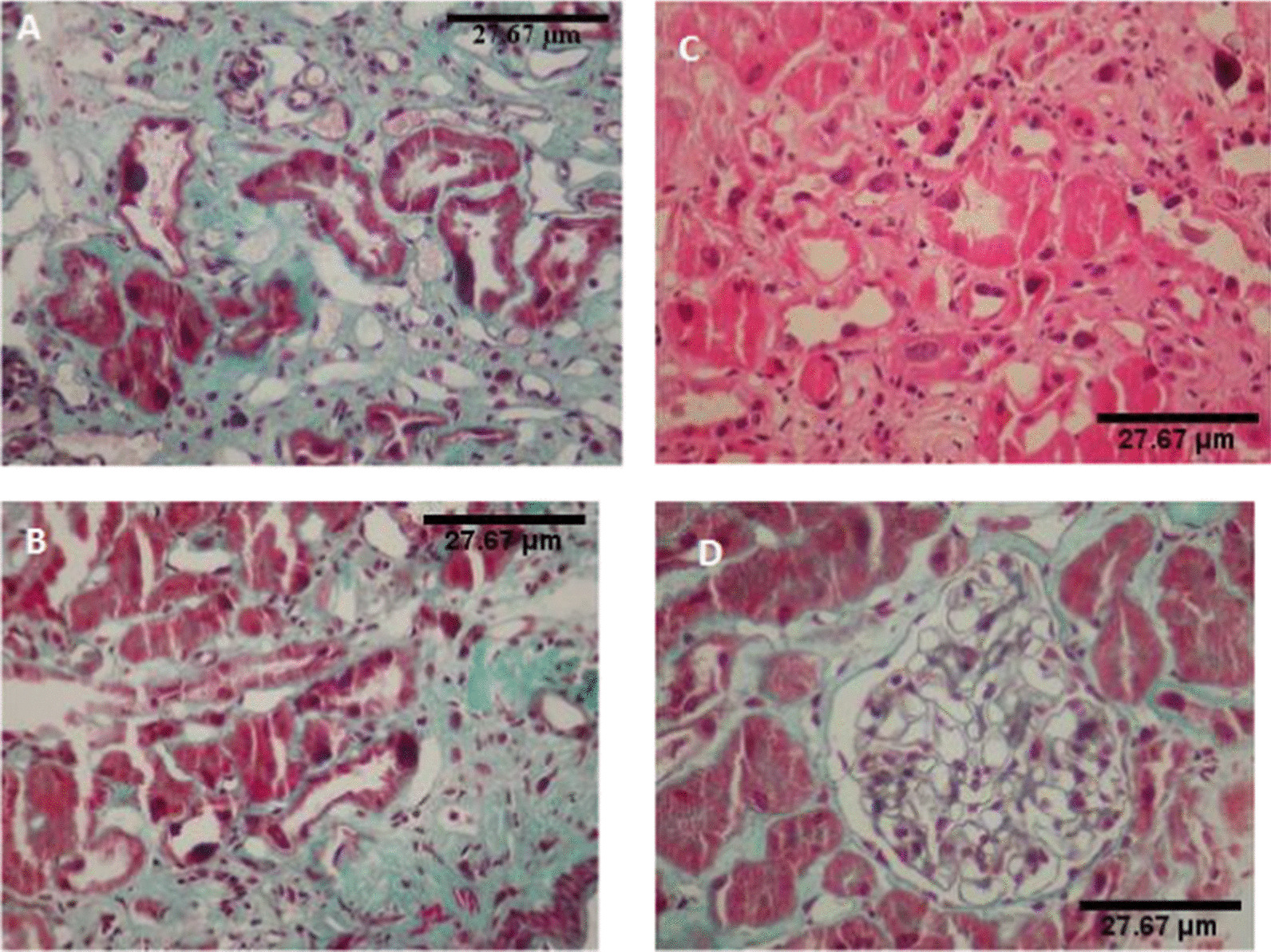
Renal histology in Patient II 3 in family Z. **a** Renal biopsy, Masson’s trichroma (magnification × 200).Giant nuclei of tubular epithelium. **b** Renal biopsy, Masson’s trichroma (magnification × 400). Giant nuclei of tubular epithelium, moderately inflammatory interstitial fibrosis. **c** Renal biopsy. Hematoxylin and eosine stain (magnification × 200). Karyomegalic nephropathy. **d** Renal biopsy. Masson’strichroma (magnification × 400). Normal glomerulus

He was admitted for a kidney donor checkup. He had a history of epilepsy and visceral leishmaniasis. Kidney failure with serum creatinine level at 200 µmol/L was discovered. Renal biopsy revealed KIN (Fig. 3).

The urine dipstick test and blood pressure of the other family members were normal. The liver function test did not reveal cytolysis nor cholestasis.

Table 1 summarizes the available laboratory parameters and clinical data.

The discovery of two members with KIN confirmed by renal biopsy (Patients IV-7 and IV-12) and two others with ESRD (Patients IV-2 and IV-11) in family A due to tubulointerstitial nephropathy in four siblings was highly suggestive of hereditary chronic interstitial nephropathy.

### Mutation screening of the *FAN1* gene by direct sequencing

Based on these clinical findings, and after written consent, sequencing of the *FAN1* gene was performed for each family proband (Patient IV-2 of family A and Patient II-3 of family Z).

Genomic DNA was extracted from peripheral blood leukocytes by the standard proteinase-K extraction.

For *FAN1* gene analysis, the coding regions (13 exons, GenBank accession number NM_014967.4) and flanking intronic sequences were amplified by PCR using genomic DNA according to the PCR protocol described by Zhou et al. [4].

PCR products were directly sequenced with the Big Dye Terminator ready reaction kit (PE Applied Biosystems) on an Applied Biosystems SeqStudio Genetic Analyzer. Base-calling was performed by using Sequencing analysis software v7.0.

The reference sequence was obtained from the UCSC Human Genome Browser (http://www.genom.ucsc.edu; *FAN1*: NM_014967.4).

The results were analyzed using Sequencher 5.0 Demo. Three programs (Polyphen-2 software, SIFT, and MutationTaster) were used to predict the functional effect of the detected variations.

## Results

**Fig. 4:**
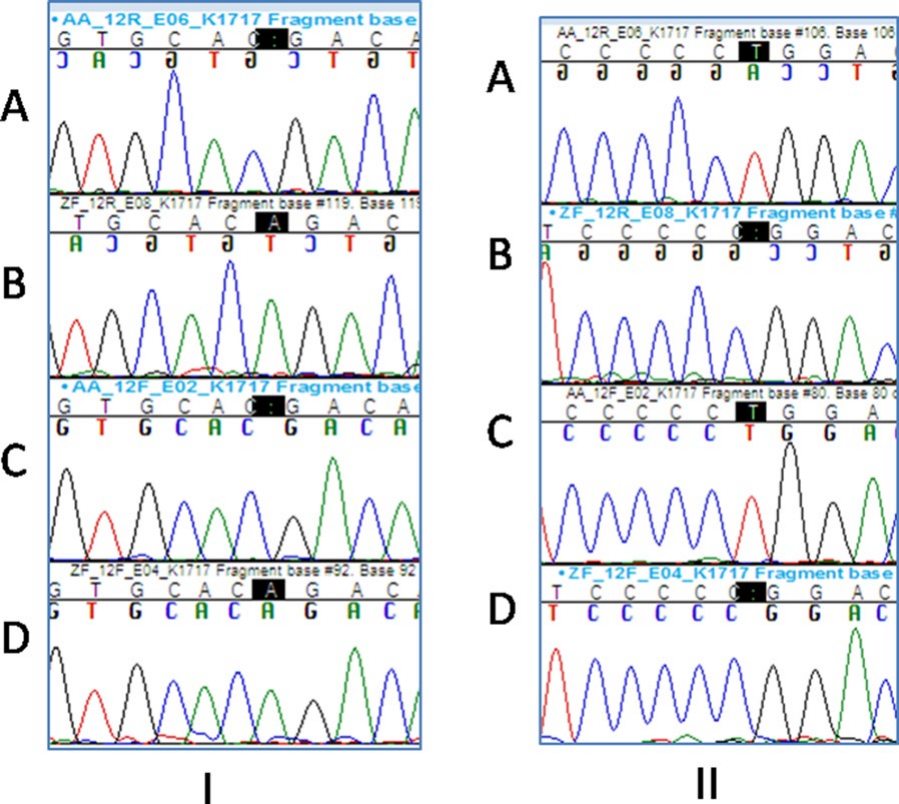
I: Electropherogram showing the mutation c.2616delA found in family A (**a**, **c** mutation found in patient IV-2, **b**, **d** wildtype). II: Electropherogram showing the mutation c.2603delT found in family Z (**a**, **c** wildtype, **b**, **d** mutation found in patient II-3)

Genetic analysis by Sanger sequencing of *FAN1* of each family proband allowed us to identify two different mutations in exon 12:

In patient IV-2 of Family A, we found a homozygous frameshift mutation due to a one-basepair deletion (c.2616delA), resulting in the appearance of a premature STOP codon (p.Asp873ThrfsTer17) (Fig. 4).

The identified mutation was tested for familial segregation in IV-5, IV-12, and IV-15. The observed mutation was found in a homozygous state in the affected brother (IV-12) and was absent in the healthy brother (IV-5). The parents of the proband and his sister (IV-15) were heterozygous for the mutation. The variation was found to segregate with the phenotype and in obligate carriers.

Regarding patient II-3 of Family Z, we found a homozygous frameshift mutation due to a one-base-pair deletion (c.2603delT), resulting in the appearance of a premature STOP codon (p. (Leu868ArgfsTer22)) (Fig. 4). This mutation was reported neither in the literature nor in online databases as polymorphism. The three prediction programs (PolyPhen2, SIFT, and MutationTaster) showed a consistent result of the deleterious effect of this mutation. The parents of the proband of Family Z were heterozygous for the mutation; the rest of the family was not tested.

## Discussion

Karyomegalic interstitial nephritis (KIN) is a rare genetic disease that was first described by Burry [1] and given this term later by Mihatsch et al. [2], who described two siblings and an unrelated third patient with progressive chronic renal failure disease.

Patients with KIN usually show progressive renal dysfunction, haematoproteinuria, and a family history of renal disease. They frequently develop recurrent respiratory infections in their 20s [2] that were not noticed in our patients.

Transient elevations of liver enzymes were observed in our patients (Table 1) as described in others [2, 8, 9].

An end-stage renal failure appears before 50 years of age [3]. In our series of patients, the mean age at first documentation of nephropathy was 26.83 years. In the literature, the average age is 36.42 years. Indeed, most patients, including ours, developed a progressive renal failure beginning in the third decade of life. The evolution to ESRD is usually at an age of 37.22 years (36.5 years in our series).

Karyomegaly is not kidney-specific. Mihatsch et al. [2], reported biopsy specimens of several organs of a patient and the autopsy of one patient that disclosed polymorphic enlarged nuclei in epithelial and mesenchymal cells of the kidneys and many other organs, i.e. smooth muscle cells of the intestine, alveolar epithelial cells, brain astrocytes, bile duct epithelium and, Kupffer cells. In our cases, one liver biopsy was performed and did not show karyomegalic cell nuclei, which suggests a predominance of renal sensitivity that is probably due to a specific repair mechanism in kidney tubular cells.

In 2012 Zhou et al. [4] identified recessive mutations in the *FAN1* gene as causing KIN. To date, to our knowledge, 12 families with an autosomal recessive inheritance have been reported with *FAN1* mutations.

By taking into account these results, we sequenced the *FAN1* gene for some of the affected members of our families. Sequencing allowed us to identify 2 different mutations in the exon 12 for each family.

The c.2616delA mutation resulting in a premature STOP codon (p.Asp873ThrfsTer17) was detected in Family A (Patients IV-7 and IV-12) (Fig. 4). This mutation was previously reported in a family with KIN by Zhou et al. [5] and recently by Dash et al. [7].

A new homozygous frameshift mutation (c.2603delT) resulting in a premature STOP codon (p.Leu868ArgfsTer22) was detected in Family Z (Patient II-3) (Fig. 4).

To the best of our knowledge, nine mutations in the exon 12 of *FAN1* gene were reported in the literature in families with KIN [4, 7], our mutations are the newest ones (Table 2).

**Table 2:** Review of mutations reported in the *FAN1* gene

References	Ethnic origin	Nucleotide alteration	Deduced protein change	Exon/intron (state)	Parental consan-guinity
Zhou et al. [4]	French	c.1234 + 2T>A c.2036_7delGA	Splice site p.Arg679Thrfs*5	2 (het) 7 (het)	No
Zhou et al. [4]	French	c.1234 + 2T>A c.2245C>T	Splice site p.Arg749*	2 (het) 9 (het)	No
Zhou et al. [4]	French	c.1375 + 1G>A c.2616delA	Splice site p.Asp873Thrfs*17	3 (het) 12 (het)	No
Zhou et al. [4]	German	c.1606C>T c.2786A>C	p.Arg536* p.Gln929Pro	5 (het) 12 (het)	No
Zhou et al. [4]	German	c.1606C>T c.2878G>A	p.Arg536* p.Asp960Asn	5 (het) 13 (het)	No
Zhou et al. [4]	New Zealand Maori	c.2120G>A	p.Trp707*	8 (hom)	distant?
Zhou et al. [4]	Spanish	c.2616delA	p.Asp873Thrfs*17	12 (hom)	No
Zhou et al. [4]	Swiss	c.2611T>C c.2878G>A	p.Cys871Arg p.Asp960Asn	12 (het) 13 (het)	No
Zhou et al. [4]	USA	c.2774_5delTT c.2810G>A	p.Leu925Profs*25 p.Gly937Asp	12 (het) 13 (het)	No
Isnard et al. [3]	French	c.2616delA	p.Asp873Thrfs*17	12 (hom)	ND
Dash et al. [6]	Bulgarian	c.1102C>T c.2616delA	p.Gln368* p.Asp873Thrfs*17	2 (het) 12 (het)	ND
Koshy et al. [5]	Indian	c.1356T>G c.1369C>T	p.Asn452Lys p.Gln457*	4(het) 4 (het)	ND
Present study Family A	Tunisian	c.2616delA	p.Thr872Thrfs*17	12 (hom)	Yes
Present study Family Z	Tunisian	c.2603delT	p.Leu868Argfs*21	12(hom)	Yes

Hom, homozygous; Het, heterozygous; ND, no data

The most recurrent mutation is c.2616delA found in French [3, 4], Spanish [4], and recently in Bulgarian [7] families (Table 2). Family A reported here is the fifth family. Zhou et al. [4] considered this mutation as a European founder mutation. In North African countries, especially in Tunisia, there is a great heterogeneity of ancestors. We can suggest that this is a founder mutation only by comparing of haplotypes between the families harboring the same mutation to distinguish whether it derives from an older or more recent single mutational event or whether it has arisen independently more than once.

*FAN1* encodes Fanconi anemia-associated nuclease 1 (FAN1), which interacts with Fanconi Anemia Complementation group D2 (FANCD2) and Fanconi Anemia Complementation group I (FANCI), forming the Fanconi Anemia core complex. This complex is recruited at the sites of Interstrand DNA crosslinks (ICL) lesions [10–13].

Zhou et al. suggest that biallelic mutations in *FAN1* cause KIN instead of FA [5]. The cause of the karyomegaly in KIN is not yet understood, but according to Lachaud et al. [14], defective ICL repair can cause karyomegaly.

Chaki et al. suggested that there is a link between degenerative kidney diseases and defective DNA damage repair [15].

More recently, Law et al. [16] reported the first case of a patient with concurrent leukocyte chemotactic factor 2 amyloidosis (ALECT2) and KIN caused by a novel deletion in *FAN1*. This highlights the importance of renal biopsy in chronic kidney disease of unclear etiology.

## Conclusions

In conclusion, our study is the first Tunisian report of familial cases of KIN with mutations involving the *FAN1* gene, which plays a crucial key role in DNA repair. The *FAN1* gene should be screened in families with more than one case with KIN. Familial counseling must be offered to the rest of the families for allograft planning.

## Data Availability

The principal datasets generated and/or analyzed during the current study are included in the published article. The datasets used in this study are available from the corresponding author by request. The sequence data are available in the NCBI SRA under the accession number PRJNA727732 (https://www.ncbi.nlm.nih.gov/Traces/study/?acc=PRJNA727732).

## Abbreviations

KIN: Karyomegalic interstitial nephritis
FAN1: FANCD2/FANCI-Associated Nuclease 1
ESRD: End-stage renal disease
eGFR: Estimated glomerular filtration rate
ANCA: Antineutrophil cytoplasmic antibody
ANA: Antinuclear antibody
FAN1: FANCD2/FANCI-Associated Nuclease 1
FANCD2: Fanconi Anemia Complementation group D2
FANCI: Fanconi Anemia Complementation group I
DDR: DNA damage response
ICL: Interstrand crosslinks
FA: Fanconi anemia
ZNF423: Zinc Finger Protein 423
CEP164: CentrosomalProtein 164
ALECT2: Leukocyte chemotactic factor 2 amyloidosis
